# Nomogram to predict risk of neonatal mortality among preterm neonates admitted with sepsis at University of Gondar Comprehensive Specialized Hospital: risk prediction model development and validation

**DOI:** 10.1186/s12884-024-06306-4

**Published:** 2024-02-15

**Authors:** Tigabu Kidie Tesfie, Degefaye Zelalem Anlay, Birhanu Abie, Yazachew Moges Chekol, Negalgn Byadgie Gelaw, Tsion Mulat Tebeje, Yaregal Animut

**Affiliations:** 1 Department of Epidemiology and Biostatistics, Institute of Public Health, College of Medicine and Health Sciences, University of Gondar, Gondar, Ethiopia; 2 School of Nursing, College of Medicine and Health Sciences, University of Gondar, Gondar, Ethiopia; 3 Department of Pediatrics and Child Health, School of Medicine, College of Medicine and Health Sciences, University of Gondar, Gondar, Ethiopia; 4 Department of Health Information Technology, Mizan Aman College of Health Science, Mizan Aman, Ethiopia; 5 Department of Public Health, Mizan Aman College of Health Science, Mizan Aman, Ethiopia; 6 School of Public Health, College of Medicine and Health Sciences, Dilla University, Dilla, Ethiopia

**Keywords:** Prediction, Mortality, Preterm neonates, Sepsis, Ethiopia

## Abstract

**Background:**

Mortality in premature neonates is a global public health problem. In developing countries, nearly 50% of preterm births ends with death. Sepsis is one of the major causes of death in preterm neonates. Risk prediction model for mortality in preterm septic neonates helps for directing the decision making process made by clinicians.

**Objective:**

We aimed to develop and validate nomogram for the prediction of neonatal mortality. Nomograms are tools which assist the clinical decision making process through early estimation of risks prompting early interventions.

**Methods:**

A three year retrospective follow up study was conducted at University of Gondar Comprehensive Specialized Hospital and a total of 603 preterm neonates with sepsis were included. Data was collected using KoboCollect and analyzed using STATA version 16 and R version 4.2.1. Lasso regression was used to select the most potent predictors and to minimize the problem of overfitting. Nomogram was developed using multivariable binary logistic regression analysis. Model performance was evaluated using discrimination and calibration. Internal model validation was done using bootstrapping. Net benefit of the nomogram was assessed through decision curve analysis (DCA) to assess the clinical relevance of the model.

**Result:**

The nomogram was developed using nine predictors: gestational age, maternal history of premature rupture of membrane, hypoglycemia, respiratory distress syndrome, perinatal asphyxia, necrotizing enterocolitis, total bilirubin, platelet count and kangaroo-mother care. The model had discriminatory power of 96.7% (95% CI: 95.6, 97.9) and *P*-value of 0.165 in the calibration test before and after internal validation with brier score of 0.07. Based on the net benefit analysis the nomogram was found better than treat all and treat none conditions.

**Conclusion:**

The developed nomogram can be used for individualized mortality risk prediction with excellent performance, better net benefit and have been found to be useful in clinical practice with contribution in preterm neonatal mortality reduction by giving better emphasis for those at high risk.

## Background

The highest risk of death in children occurred in their first month of life. Globally, 2.4 million children died in their neonatal period in 2020 which was approximately 6,500 neonatal deaths every day. Up on this figure nearly half (47%) of all under-five deaths occurred in the first 28 days of life [1]. The first 28 days of life from birth is the most critical time of human life for diseases and death [2].

Neonatal mortality is highly disproportional in developed and developing countries. In developed countries, neonatal mortality average around 3 per 1000 live births in contrast it is around 26 per 1000 live births in developing countries [3]. Sub-Saharan Africa(SSA) has the highest neonatal mortality rate in the world (27 deaths per 1000 live births) with 43% of global newborn deaths, followed by the central and southern Asia (23 deaths per 1000 livebirths), with 36% of global newborn deaths [4]. According to the Ethiopian demographic and health survey reports, the rate of neonatal mortality fell gradually from 48.7 deaths per 1,000 live births in 2000 to 30 deaths per 1,000 live births in 2019 [5]. Ethiopia is among countries that comprise 50% of global neonatal mortality with India (24%), Pakistan (10%), Nigeria (9%) and Congo (4%) [6].

Neonatal sepsis and preterm birth are the leading causes of global neonatal mortality [4, 7]. The co-occurrence of preterm birth and sepsis makes the newborn more vulnerable for mortality. The case fatality rate of sepsis in premature neonates reaches up to 20% which is disproportionately higher than term neonates [8, 9]. Approximately 15 million preterm birth occur each year throughout the world from these at least one million of them die due to complications of prematurity including sepsis [3].The high level of mortality among septic preterm neonates have a long-term consequences on national and international economic development [4, 7].

The world has made extensive progress in neonatal mortality reduction in the last three decades. Worldwide, the number of neonatal deaths reduced from five million in 1990 to 2.4 million in 2020 (in thirty years). However, this decline in neonatal mortality has been slower than that of post neonatal under-five mortality [4]. The health status of premature infants who have sepsis is closely related to the quality of maternal and neonatal health care which is poor in developing countries [10]. Very slow improvements in quality of antenatal and perinatal care including failure to make early predictions play role in little achievements of neonatal mortality reduction in developing countries.

To reduce mortality rate of neonates, every newborn action plan (ENAP) was drafted in 2014 with its first goal, in all countries to reach the target of 12 or less and 10 or less newborn deaths per 1000 live births to be achieved by 2030 and 2035 respectively [11]. The United Nations (UN) sustainable development agenda to be achieved by 2030 goal-three (SDG-3) focus on ensuring healthy lives and promote well-being for all at all age [12]. The rate of neonatal mortality in Ethiopia is still unacceptably high.

Several factors were previously identified as predictors of neonatal mortality. The most common factors are: sex [13–16], age [6, 17, 18], gestational age (GA) [13, 19], admission weight [6], weight for gestational age (WGA) [20], Apgar score at first and fifth minute [13, 21], initiation of breast feeding timing [19, 22, 23], kangaroo mother care (KMC) [22, 23], premature rupture of membrane(PROM) [13] and antenatal steroid [13, 21] and comorbidities including perinatal asphyxia (PNA) [24–26], respiratory distress syndrome (RDS) [23, 27–31], necrotizing enterocolitis (NEC) [32] and hypoglycemia [6].

Even if neonatal mortality in preterm neonates with sepsis is high, it is less researched regarding development of prognostic model. To our knowledge, there are no risk assessment tools developed so far for preterm septic neonates to predict their mortality risk. This study would have contribution in reduction of mortality through early prediction. Prediction models help healthcare professionals and patients to make clinical decisions through providing patient risk stratification to support tailored clinical decision-making with the hope of improving patient outcomes and quality of care [33].

The aim of this study was to develop easy to use, less resource intensive and biologically plausible clinical prediction model (nomogram) applicable for every preterm neonate with sepsis. This study would be the first in our setting to predict mortality with risk prediction model in preterm septic neonates. Up to our knowledge, there is no study on the risk prediction of preterm neonatal mortality with sepsis in Ethiopia. A predictive model allows timely preterm septic neonates risk stratification, which guides the clinical care for better health outcome of the neonates. This model would help physicians in directing the management process by considering the risk of the individual for mortality. This had role in the reduction of neonatal mortality and contribute to SDG 3.2 and ENAP goal-1 through improvement of the decision making process in the course of treatment.

## Methods

### Study design and setting

An institution based retrospective follow up study was conducted at University of Gondar Comprehensive Specialized Hospital from January 1st, 2019 to December 31st, 2021. Data extraction was made from May 30, 2022 to June 23, 2022. University of Gondar Comprehensive Specialized Hospital is one of the largest teaching hospitals in Ethiopia found in Amhara region providing tertiary level care for more than seven million people in the northwest part of the country. The hospital is found in Gondar which is located at 740 and 180 km far from Addis Ababa and Bahir Dar, the capital city of Ethiopia and Amhara region to northwest respectively. The neonatal intensive care unit (NICU) is included under the pediatrics and child health department, provides medical care for an inpatient neonates. It has a caring capacity of about 42 beds at a time, 12 of these beds are for preterm neonates. There are sixteen incubators in the unit.

### Population

All preterm neonates with sepsis admitted at University of Gondar Comprehensive Specialized Hospital were the source population, while those admitted from January 2019 to December 2021 were the study population. This study included preterm neonates admitted with sepsis at University of Gondar Comprehensive Specialized Hospital from January 1st, 2019 to December 31st, 2021 and preterm neonates with sepsis died on arrival, disappeared from medical treatment and with incomplete record on outcome were excluded. In preterm neonates died on arrival, it is difficult to assess their prognostic determinants and to make prediction on their mortality risk since there is no time difference between predictor and outcome assessment hence they were excluded from the study. Neonates disappeared from medical treatment are also with unknown outcome status. Since it is difficult to develop prognostic model based on samples that have unknown outcome status, neonates with incomplete record on outcome were excluded.

### Sample size determination and sampling procedure

#### Sample size determination

During sample size calculation for this multivariable prognostic model we used the rule of thumb method of ten events per parameter. This sample size calculation method was most widely used in previous prognostic studies to minimize prediction error and problem of overfitting [34, 35]. According to a study done at Felege-Hiwot Specialized Hospital, the prevalence of preterm neonatal mortality among sepsis cases was 44.7% [36]. The minimum sample size was calculated considering assumptions of 10 events per parameter (EPP), the proportion of mortality among preterm neonates with sepsis $(\phi =0.447)$ from previous study and 25 predictors (P). Sample size was calculated [37] as follows;

$${\varvec{n}}=\frac{{\varvec{E}}{\varvec{P}}{\varvec{P}}\boldsymbol{*}{\varvec{p}}}{{\varvec{\phi}}}$$

$${\varvec{n}}=\frac{{\varvec{E}}{\varvec{P}}{\varvec{P}}\boldsymbol{*}{\varvec{p}}}{{\varvec{\phi}}} =\frac{10\boldsymbol{*}25}{0.447}\boldsymbol{ }= 560$$

After adding 10% (56 samples) contingency for missing chart, the final sample size was 616.

*Where;* EPP: Events per parameter, P: number of predictors and $\phi$: proportion of neonatal mortality in preterm neonates with sepsis from previous research.

#### Sampling procedure

**Fig. 1:**
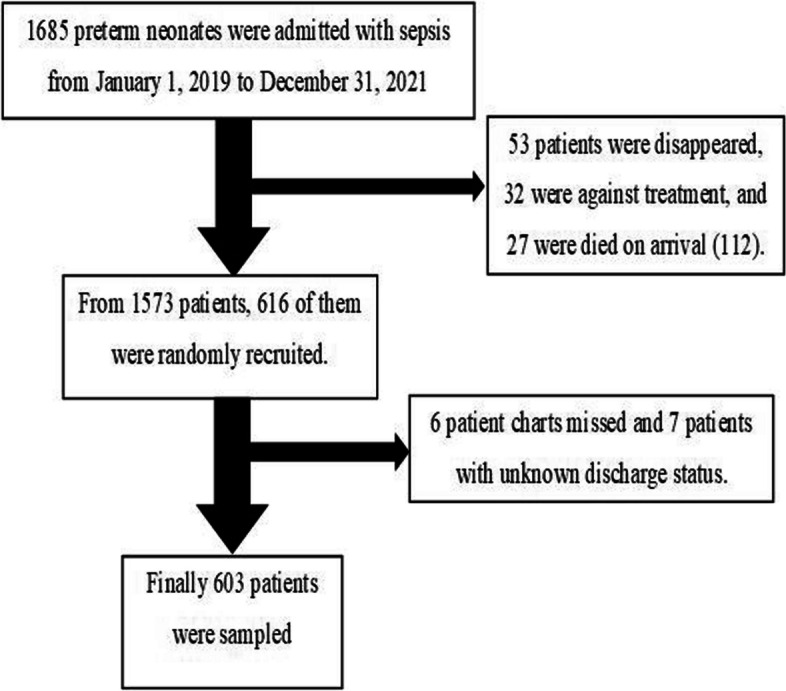
Participant flowchart for prediction of neonatal mortality in preterm neonates admitted with sepsis at University of Gondar Comprehensive Specialized Hospital, January 2019 to December 2021

Simple random sampling technique using computer generated random numbers was employed in this study. The number of preterm neonates admitted with sepsis with in the three years follow up period identified from the registration book were 1685. Out of the total admitted participants, 112 were excluded using their registration book (53 patients were disappeared, 32 were against treatment, and 27 were died on arrival). Then the medical record numbers of 1573 preterm neonates were recorded using Microsoft Excel and a total of 616 random samples were generated. Then 6 charts were missed and seven charts were with unknown discharge status (outcome not available). Finally 603 participants were included in the study (Fig. 1).

### Variables of the study

The outcome variable was mortality among preterm neonates admitted with sepsis (yes/no). Prognostic determinants were: Sex, age, referral from other health facility, gestational age, admission weight, weight for gestational age and Apgar score at 1st and 5th minute, initiation of breast feeding, kangaroo-mother care, resuscitation, perinatal asphyxia, respiratory distress syndrome, necrotizing enterocolitis and hypoglycemia, platelet count, serum total bilirubin, hemoglobin level, mode of delivery, parity, antenatal care visits, hypertensive disorder of pregnancy, antepartum hemorrhage, premature rupture of membrane and antenatal steroid.

### Operational definitions

#### Neonatal mortality

In this study implies death of a live born preterm neonate admitted with sepsis within the first 28 days of life [38]. A preterm septic neonates whose discharge status documented by the attending clinician as “died” from their chart were considered as “died” and those preterm septic neonates with improved or cured discharge status were considered as “not died” during outcome ascertainment.

#### Sepsis

A documented physician diagnosis of sepsis. The physician diagnosis is made based on the neonate presentation with any one of the systemic manifestations of neonatal danger signs [39] and or laboratory confirmation of sepsis through blood culture.

#### Perinatal Asphyxia

A documented physician diagnosis perinatal asphyxia following the neonate inability to initiate and sustain breathing at birth with < 7 Apgar score for longer than 5 min [65].

#### Necrotizing enterocolitis

A documented physician diagnosis of necrotizing enterocolitis by the following findings of fixed and dilated intestinal loop that does not change on repeated x-ray with signs of pneumatosis intestinalis, thickened bowel wall, pneumoperitoneum and portal vein gas and detection of blood in the stool [39].

#### Hypoglycemia

A documented physician diagnosis of hypoglycemia based on decreased level of random blood sugar (below 50 mg/dl) detected by glucometer.

### Data collection procedures and quality control

Based on different literatures, structured data extraction checklist was prepared. The checklist included demographic and birth characteristics of the neonate, newborn interventions and diagnosed comorbidities, laboratory profile of the neonate and maternal obstetric factors. Then electronic data collection form was prepared with Kobo Toolbox software online and data was collected with KoboCollect version 2022.1.2 application using android version mobile phones. Training about: the objective of the study, data extraction and confidentiality of the participant’s information, submission of collected data to the box and use of tools was given to data collectors (three nurses) and the supervisor (one health officer) for one day. Before the actual data collection, preliminary chart review was done on 5% of the study participants and appropriate correction was made on the extraction checklist. Data cleaning, coding, recoding and missing data handling was done accordingly to improve data quality.

### Data processing and analysis

The data collected with KoboCollect, was exported to STATA/MP version 16 and R version 4.2.1 software for management and further analysis. Majority of the analysis was conducted using STATA but the prediction density plot and decision curve analysis were conducted using R. Descriptive summary statistics was carried out accordingly. Frequencies with percentages were presented for categorical variables. The analysis and reporting of study results was done according to the Transparent Reporting of a multivariable prediction model for Individual Prognosis Or Diagnosis (TRIPOD) Initiative checklist [40].

### Missing data management

Out of the candidate predictors’, thirteen variables were with missing observations. The proportion of missing in the collected data was described with frequency and percentage using the *“mdesc”* package in STATA. Variables with missing values were: weight for gestational age 10 (1.7%), Apgar score at first minute 13 (2.2%), Apgar score at fifth minute 13 (2.2%), number of ANC visits 4 (0.7%), preterm PROM 16 (2.7%), antenatal steroid 17 (2.8%), platelet count 36 (6.0%), hemoglobin level 37 (6.1%), total serum bilirubin 40 (6.6%), hypoglycemia 11 (1.8%), breast feeding initiation 19 (3.2%), kangaroo-mother care 50 (8.3%) and resuscitation 112 (18.6%). Multiple imputation was done using the “*mi impute chained”* command using random number seeds. Sensitivity analysis was done after multiple imputation and showed agreement with the original data but the imputed data had the advantage of better sample (the power of the study was kept) [41].

### Model development and validation

The theoretical design of the study was; the incidence of mortality at a future time (“*tf*”) as a function of multiple prognostic predictors ascertained during admission (i.e., “t_0_”, the moment of prognostication) in a domains of preterm neonates admitted with sepsis. The occurrence function of the study was mortality as a function of predictors’* f* (gestational age + preterm premature rupture of membrane + hypoglycemia + respiratory distress syndrome + perinatal asphyxia + necrotizing enterocolitis + total bilirubin + platelet count + kangaroo-mother care). Formal model development was begin with the identification of predictors after data cleaning and generating imputed datasets. Well-performing prediction model requires some number of strong predictors being present to increase applicability in a clinical practice [42]. We performed least absolute shrinkage and selection operator (LASSO) regression analysis to select the most potent predictors. Then the best and optimum lambda was fitted using tenfold cross-validation. Then variables with non-zero coefficients from LASSO with the optimum cross-validated lambda and smallest cross validated mean deviance result was used to fit multivariable binary logistic regression model [43]. Multicollinearity was checked through Variance Inflation Factor (VIF), multicollinearity was not observed between predictors (the maximum VIF observed was 1.47 and mean VIF was 1.25). Then model reduction was done to develop more simplified and easy to use individualized risk prediction model, through assessing the role of predictors by reducing one by one from the full multivariable model at a probability value of 0.15 using likelihood ratio test (models compared were nested). Then the final risk prediction model was developed in the form of nomogram to graphically depict a statistical prognostic model that generates a probability of mortality for a given preterm neonate with sepsis based on the retained variables from the reduced model using with p-value of less than 0.05. Nomogram helps to improve the applicability of a multivariable model in practice, maintaining the original regression coefficients unchanged as compared to risk score and it is user friendly graphical presentation of prognostic models [44]. Nomogram was constructed using “nomolog” STATA package [45].

Model performance was evaluated by considering the difference between the predicted outcome and the actual outcome using the two characteristics of performance; calibration and discrimination [33]. Calibration was assessed visually by plotting the observed probability of mortality (y-axis) against the predicted probability of mortality (x-axis), with perfect predictions falling along a 45° line. Statistical summary measure of calibration which is the Hosmer and Lemeshow test was assessed [46] in order to evaluate the agreement between the observed and expected mortality rates across different risks thresholds. Insignificant probability value of goodness of fit test indicates the model similar performance across different risk categories in the observed and expected probabilities [37]. Discrimination is the ability of a model to distinguish individuals who died from those who remained death free [33]. We assessed the nomogram discriminatory power using the concordance statistics (c-statistic). The c-index is equal to the area under the receiver operating characteristic curve (AUC), and ranges between 0.5–1. A c-index of 1 indicates a model that is perfectly discriminating, and a value of 0.5 indicates inability of the model to discriminate between these two groups [46, 47]. Brier score, the proper and strict measure of model overall accuracy was assessed. Brier score approaches to 0 indicates the better prediction accuracy but when it approaches to 1 indicates the worst model accuracy [46]. Mortality risk classification was done after identifying the cutoff point by Youden’s J statistics. The classification was made as low, and high risk of mortality. The corresponding proportions were calculated for low risk and high risk groups. Additionally performance of developed nomogram was assessed using sensitivity, specificity, positive predictive value and negative predictive value with their respective confidence intervals at the optimum cutoff point.

Parametric bootstrapping was performed for internal validation based on random number seeds. Bootstrapping method of internal validation is an ideal method for smaller sample sizes or for larger numbers of candidate predictors [33, 37]. Bootstrapping aims to repeated with replacement within the study population to create multiple training subsets [46]. One thousand bootstrap samples were generated and returned to the pool (through sampling with replacement) [48]. Model performance was assessed after bootstrapping to observe the degree of over fitted model by comparing with the original model performance. No difference (zero optimism coefficient) was observed between the apparent performance (actual model performance on samples) and true performance (model performance of after internal validation).

### Net benefit analysis

Standardized net benefit was assessed through decision curve analysis, which allows to understand the implications of basing decisions to operate on the predictions generated from the risk prediction model across a range of predicted risks to be compared using a common scale. Decision curve analysis was performed using R software with *rmda* and *DCA* packages to assess the net benefit of using the developed model by clinicians in a clinical practice [49]. The cost and benefit ratio was analyzed across threshold probabilities.

## Results

### Neonatal demographic and birth characteristics

A total of 603 patients were included in the study producing a 97.9% completeness rate. Out of all participants, more than half 355 (58.9%) of them were males. Majority of the neonates 526 (87.2%) were admitted at or within their first day of life after birth. Regarding referral status, 225 (37.3%) of neonates were referred from other health facility. About 170 (28.2%) neonates were delivered before 32 completed weeks of gestational age. Most of neonates 557 (92.4%) were admitted with weight of below 2500 g. More than four-fifth 511 (84.7%) of the neonates were delivered being small for their gestational age. The first and fifth minute Apgar score were seven and above in 498 (82.6%) and 559 (92.7%) of neonates respectively (Table 1).

**Table 1:** Neonatal demographic and birth characteristics (*n* = 603)

Variables	Category	Discharge status		Total (%)
		**Died**	**Not died**	
Sex	Male	118	237	355 (58.9)
	Female	102	146	248 (41.1)
Age at admission (days)	≤ 1	193	333	526 (87.2)
	2–28	27	50	77 (12.8)
Referral from other health facility	Referred	109	116	225 (37.3)
	Not referred	111	267	378 (62.7)
Gestational age (completed week)	< 32 weeks	126	44	170 (28.2)
	> = 32 weeks	94	339	433 (71.8)
Admission weight (grams)	< 2500	208	349	557 (92.4)
	> = 2500	12	34	46 (7.6)
Weight for gestational age	SGA	67	25	92 (15.3)
	AGA	153	358	511 (84.7)
Apgar at 1st minute	< 7	73	32	105 (17.4)
	7–10	147	351	498 (82.6)
Apgar at 5th minute	< 7	32	12	44 (7.3)
	7–10	188	371	559 (92.7)

*SGA* Small for gestational age, *AGA* Appropriate for gestational age

### Newborn interventions and diagnosed medical comorbidities

Regarding the newborn interventions given at birth 428 (70.6%), 433 (71.8%) and 556 (92.2%) of admitted preterm septic neonates were initiated breast feeding after the first hour of birth, not received kangaroo-mother care and not resuscitated respectively. During admission, 89 (14.8%) of neonates were asphyxiated; 274 (45.4%) were diagnosed with respiratory distress syndrome; 36 (6.0%) of neonates were had necrotizing enterocolitis and 149 (24.7%) were hypoglycemic (Table 2).

**Table 2:** Newborn interventions and diagnosed medical comorbidities for prediction of mortality (*n* = 603)

Variables	Category	Discharge status		Total (%)
		**Died**	**Not Died**	
Breast feeding initiation	Within first hour	15	162	177 (29.4)
	After first hour	205	221	426 (70.6)
Kangaroo-mother care	Received	12	158	170 (28.2)
	Not received	208	225	433 (71.8)
Resuscitation	Resuscitated	19	28	47 (7.8)
	Not resuscitated	201	355	556 (92.2)
Perinatal asphyxia	Yes	68	21	89 (14.8)
	No	152	362	514 (85.2)
Respiratory distress syndrome	Yes	177	97	274 (45.4)
	No	43	286	329 (54.6)
Necrotizing enterocolitis	Yes	26	10	36 (6.0)
	No	194	373	567 (94.0)
Hypoglycemia	Yes	107	42	149 (24.7)
	No	113	341	454 (75.3)

### Baseline laboratory profile and maternal obstetric characteristics

Nearly half 289 (47.9%) of septic preterm neonates had a platelet count below 150 × 10^3^ per microliter. More than half 322 (53.4%) of participants were with a total serum bilirubin of 15 mg per deciliter and above. Nearly one-fifth 104 (17.2%) of neonates were with hemoglobin value of below 12 g per deciliter. In the maternal obstetric characteristics, three-fourth 459 (76.1%) of neonates were delivered with spontaneous vaginal delivery. About 267(44.3%) of mothers had less than four antenatal care visits. Fourteen percent of mothers were diagnosed with hypertensive disorders during pregnancy period of the studied neonate. Only 44 (7.3%) and 197 (32.7%) of mothers were diagnosed to have antepartum hemorrhage and preterm premature rupture of membrane respectively (Table 3).

**Table 3:** Baseline laboratory profile and maternal obstetric characteristics (*n *= 603)

Variables	Category	Discharge status		Total (%)
		**Died**	**Not Died**	
Platelet count (× 10^3^/mcL)	< 150	180	109	289 (47.9)
	≥ 150	40	274	314 (52.1)
Total serum bilirubin	< 15 mg/dl	74	207	281 (46.6)
	≥ 15 mg/dl	146	176	322 (53.4)
Hemoglobin	< 12 gm/dl	71	33	104 (17.2)
	≥ 12 gm/dl	149	350	499 (82.8)
Mode of delivery	SVD	175	284	459 (76.1)
	CS	45	99	144 (23.9)
Parity	Primipara	92	151	243 (40.3)
	Multipara	128	232	360 (59.7)
Number of ANC visits	< 4	146	121	267 (44.3)
	≥ 4	74	262	336 (55.7)
HDP	Yes	37	48	85 (14.1)
	No	183	335	518 (85.9)
Antepartum hemorrhage	Yes	23	21	44 (7.3)
	No	197	362	559 (92.7)
Preterm PROM	Yes	96	101	197 (32.7)
	No	124	282	406 (67.3)
Took antenatal steroid	Yes	16	60	76 (12.6)
	No	204	323	527 (87.4)

*mcL* Microliter, *mg/dl* Milligram per deciliter, *gm/dl *Gram per deciliter, *PROM *Premature rupture of membrane, *SVD* Spontaneous vaginal delivery, *CS* Cesarean section, *ANC* Antenatal care, *HDP* Hypertensive disorders of pregnancy

### Incidence of mortality among septic preterm neonates

**Fig. 2:**
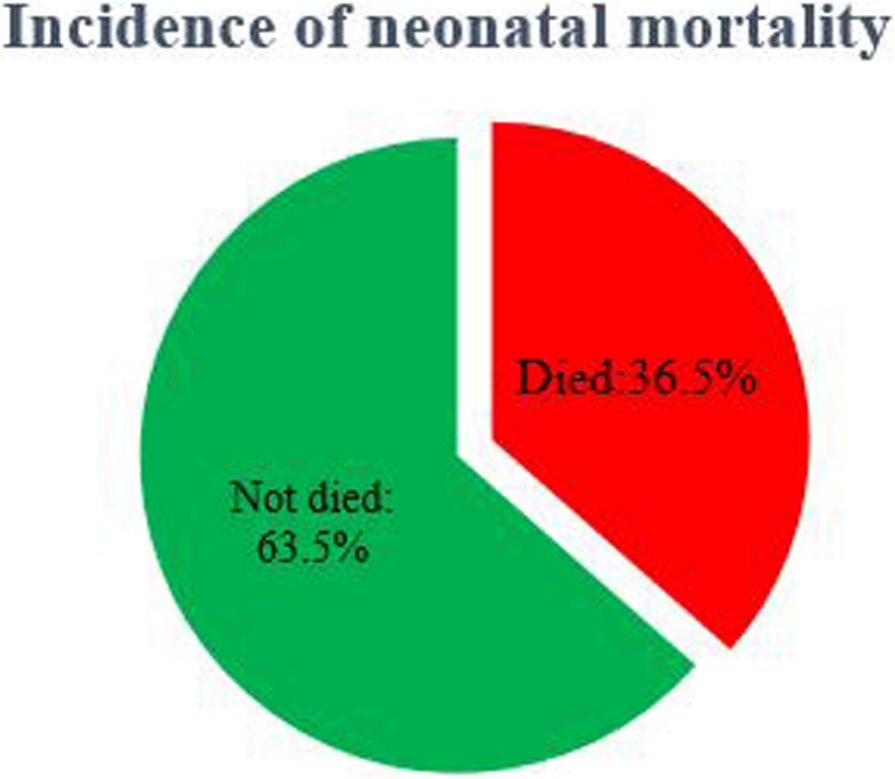
Cumulative incidence of neonatal mortality

Among the total of 603 preterm neonates admitted with sepsis, 220 (36.5% (95% CI: 32.7, 40.4)) were died (Fig. 2).

### Model development and internal validation

#### Predictor selection

After review of literatures, 25 predictors (demographic and birth characteristics, newborn interventions and diagnosed clinical comorbidities, baseline laboratory profiles of the neonate and maternal obstetric characteristics) were considered to predict mortality in preterm neonates admitted with sepsis. LASSO regression was applied and 21 predictors were selected at optimum cross validated lambda. The variables included in the LASSO regression were; sex, age, referral status, gestational age, admission weight, weight for gestational age, Apgar at first and fifth minutes, mode of delivery, parity, number of ANC visits, hypertensive disorders of pregnancy, antepartum hemorrhage, preterm PROM, antenatal steroid, perinatal asphyxia, respiratory distress syndrome, necrotizing enterocolitis, hypoglycemia, platelet count, total serum bilirubin, hemoglobin, timing of breast feeding initiation, kangaroo-mother care and resuscitation. The penalized regression reduced first minute Apgar score, antenatal steroid, resuscitation status and admission weight. The tuning parameter lambda (optimum shrinkage factor) was 0.00245 and accompanied with larger out of sample deviance ratio and smallest cross validated prediction error (Table 4).

**Table 4:** LASSO regression model using cross validation selection method (*n* = 603, covariates = 25, number of CV folds = 10)

ID	Description	lambda	Number of nonzero coefficients	Out-of-sample deviance ratio	CV mean deviance
1	first lambda	0.2565692	0	0.0018	1.309937
50	lambda before	0.0026879	24	0.6700	0.4330572
*51	**selected lambda**	**0.0024491**	**21**	**0.6700**	**0.4329958**
52	lambda after	0.0022315	21	0.6700	0.4330921
56	last lambda	0.0015381	21	0.6691	0.4342246

#### A prediction model for mortality using original β coefficients

Variables with nonzero coefficients from LASSO regression were included in the multivariable binary logistic regression analysis. Further model reduction was done by reducing each variables one by one with *p*-value of greater than or equal to 0.15 using likelihood ratio test since the models were nested. The full model (a model with 21 predictors) and a final reduced model likelihood ratio test implied no statistical difference between the two models (Likelihood Ratio *X*^2^ = 4.32 and *p*-value: 0.743). Seven variables reduced from the full model (model-1) at *P*-value of ≥ 0.15 were; weight for gestational age, mode of delivery, parity, hypertensive disorders of pregnancy, antepartum hemorrhage, hemoglobin and timing of breast feeding initiation. Finally fourteen predictors were retained and nine of them were declared as significant predictors of mortality at *p*-value of < 0.05 in the reduced model (model-2). Those retained significant predictors were; gestational age(< 32 completed weeks), preterm PROM(yes), perinatal asphyxia (yes), respiratory distress syndrome (yes), necrotizing enterocolitis (yes), hypoglycemia (yes), platelet count (< 150,000/microliter), total serum bilirubin (≥ 15mg/dl) and kangaroo-mother care (not-received) (Table 5).

**Table 5:** Multivariable binary logistic regression analysis (*n* = 603)

Predictors	Full model (Model-1)		Reduced model (Model-2)	
	**β (95% CI)**	***p*-value**	**β (95% CI)**	***p*-value**
Sex (female)	0.71 (-0.01, 1.42)	0.062	0.81 (0.11,1.52)	0.058
Age (2–28 days)	1.59 (0.38, 2.79)	0.074	1.62 (0.50, 2.74)	0.065
Referral status (referred)	0.81(-0.01, 1.62)	0.067	0.86(0.12, 1.61)	0.062
GA (< 32 weeks)	2.00 (1.14, 2.86)	< 0.001	2.26 ( 1.44, 3.09)	< 0.001
Weight for gestational age (SGA)	0.50 (-0.48, 1.48)	0.316		
Apgar at 5th minute (< 7)	1.18 (-0.27, 2.63)	0.112	1.33 (-0.12, 2.78)	0.071
Mode of delivery (CS)	-0.16 (-1.05, 0.72)	0.715		
Parity (Primipara)	0.02 (-0.71, 0.75)	0.949		
Number of ANC visits (< 4)	0.52 (-0.20, 1.24)	0.160	-0.54(-1.22, 0.13)	0.116
HDP (Yes)	0.17 (-0.85, 1.18)	0.748		
Antepartum hemorrhage (yes)	-0.07 (-1.47, 1.32)	0.918		
Preterm PROM (yes)	1.34 (0.52, 2.15)	0.001	1.25 (0.49, 2.00)	0.001
PNA (yes)	1.72 (0.73, 2.70)	0.001	1.75 (0.77, 2.73)	< 0.001
RDS (yes)	2.81 (1.86, 3.75)	< 0.001	2.87 (1.95, 3.79)	< 0.001
NEC (yes)	1.62 (0.29, 2.96)	0.017	1.89 (0.62, 3.17)	0.004
Hypoglycemia (yes)	2.48 (1.47, 3.48)	< 0.001	2.72 (1.79, 3.66)	< 0.001
Platelet count (< 150 × 10^3^/mcL)	2.07(1.32, 2.82)	< 0.001	2.12 (1.42, 2.83)	< 0.001
Total serum bilirubin (≥ 15 mg/dl)	1.71 (0.96, 2.46)	< 0.001	1.72 (1.00, 2.44)	< 0.001
Hemoglobin (< 12 gm/dl)	0.36(-0.62, 1.34)	0.474		
Breast feeding (after first hour)	0.64(-0.31, 1.60)	0.187		
KMC (not received)	3.17 (1.97, 4.38)	< 0.001	3.49 ( 2.31, 4.67)	< 0.001
Constant	-10.87 (-13.13, -8.61)	< 0.001	-10.27 (-12.44,-8.09)	< 0.001

β is coefficient of predictors in the regression model

Based on the regression coefficients of predictors in the reduced model, the equation for the multivariable prediction model was developed using coefficients.

Estimated risk of mortality (Y = Died) = 1/ (1 + exp − (− 10.27 + 2.26 × GA (< 32 weeks) + 1.25 × preterm PROM (yes) + 1.75 × PNA (yes) + 2.87 × RDS (yes) + 1.89 × NEC (yes) + 2.72 × hypoglycemia (yes) + 2.12 × platelet count (< 150,000/microliter) + 1.72 × total serum bilirubin (≥ 15 mg/dl) + 3.49 × KMC (not received)).

#### Nomogram for prediction of mortality

**Fig. 3:**
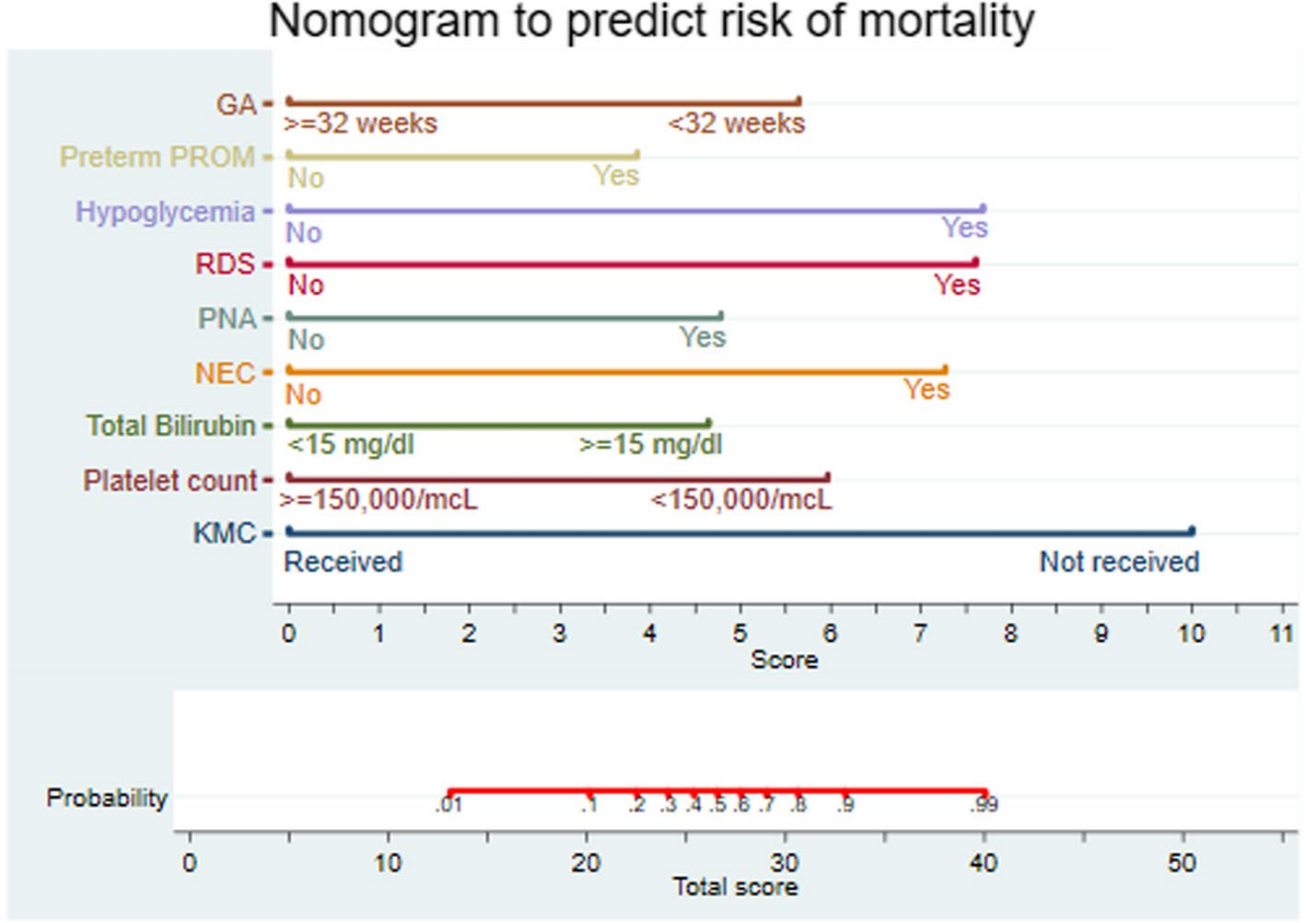
Nomogram developed for the prediction of mortality in preterm neonates admitted with sepsis at University of Gondar Comprehensive Specialized Hospital, January 2019 to December 2021

Using the final reduced model original regression coefficients, nomogram was developed for practical applicability based on predictors which have biological plausible relationship with mortality, easy to interpret, easily ascertainable and with better predictive performance. Then nine predictors were used to develop nomogram. Those predictors were; GA, preterm PROM, hypoglycemia, RDS, PNA, NEC, total bilirubin, platelet count and kangaroo-mother care. The role of each predictors in the prediction process was also presented in the form of nomogram division score (Table 6). Based on the total score the preterm neonate have, the probability of mortality could be calculated by the developed nomogram (Fig. 3).

**Table 6:** Nomogram division score for the prediction of mortality in preterm neonates with sepsis, January 2019 to December 2021

Variable	Category	Score
Gestational age	≥ 32 weeks	0
	< 32 weeks	5.6
Preterm PROM	No	0
	Yes	3.9
Hypoglycemia	No	0
	Yes	7.7
RDS	No	0
	Yes	7.6
PNA	No	0
	Yes	4.8
NEC	No	0
	Yes	7.3
Total bilirubin(mg/dl)	< 15	0
	≥ 15	4.6
Platelet count (per microliter)	≥ 150 × 10^3^	0
	< 150 × 10^3^	6.0
Kangaroo mother care	Yes	0
	No	10.0

**Fig. 4:**
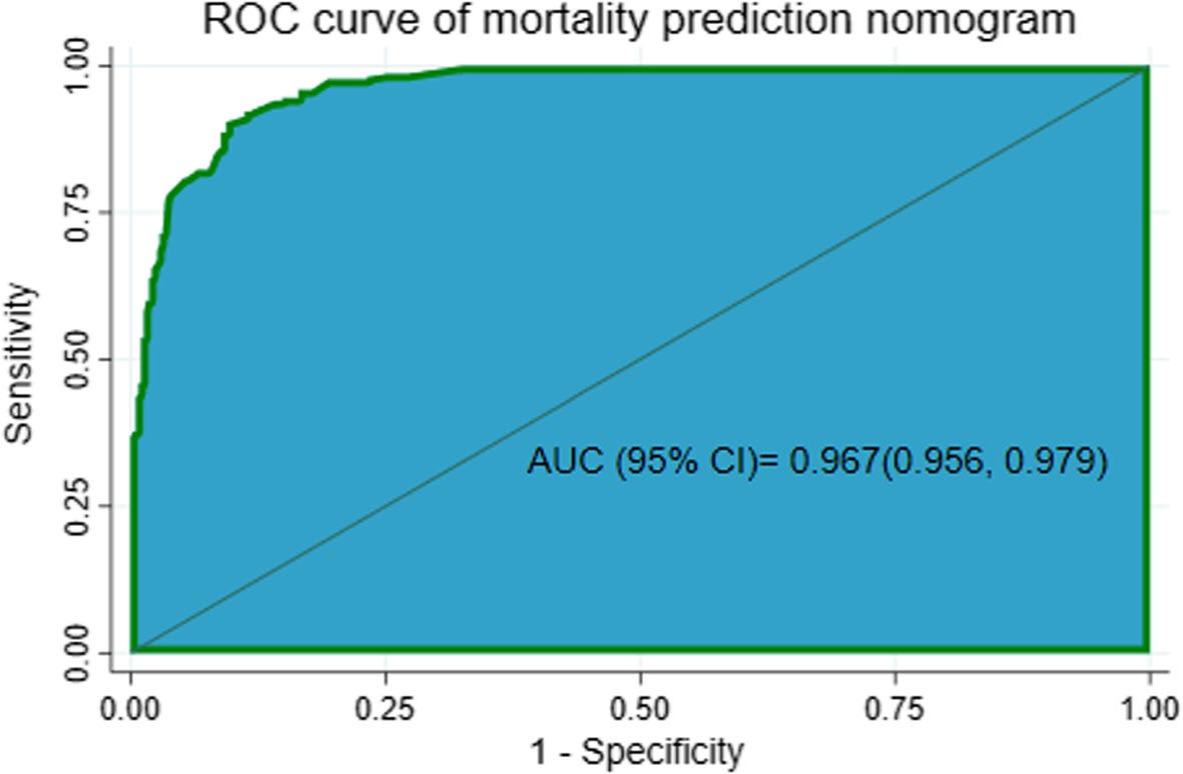
Area under the ROC curve for mortality prediction nomogram

**Fig. 5:**
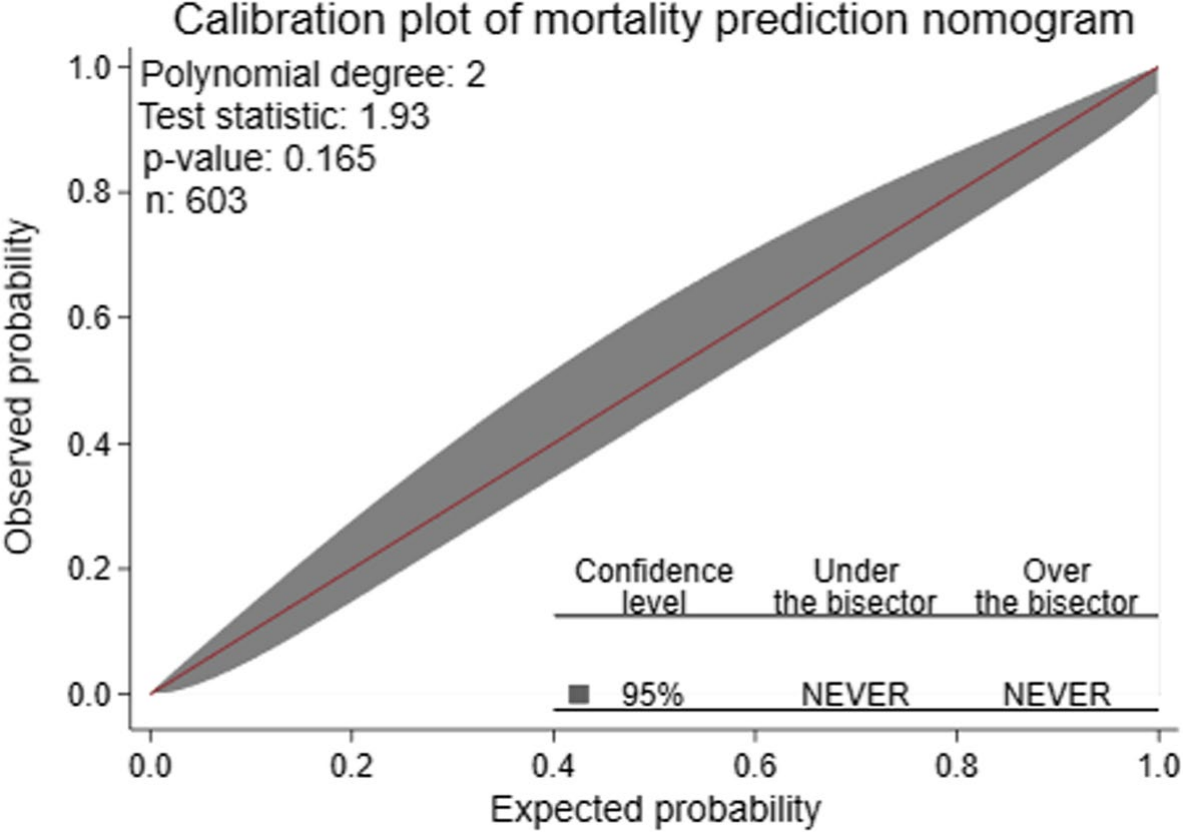
Calibration plot showing predicted (x-axis) versus observed (y-axis) probability of mortality. The bisector (red line) stands for the perfect agreement between observed and expected probability; at 95% confidence level, the calibration belt (grey color shaded region) encompass the bisector over the whole range of the predicted probabilities (the belt wasn’t observed under or over the red line). This suggests that the predicted probabilities estimated by the nomogram didn’t significantly deviate from the observed probability (that is, the model’s calibration is acceptable). Hence, at 95% confidence level, the calibration belt was neither under nor over the bisector (never under and over the bisector)

The AUC of the developed nomogram for individualized mortality risk prediction was 96.7% (95% CI: 95.6, 97.9) (Fig. 4).When we assess additional performance measures, the nomogram had sensitivity of 90.5% (95% CI: 85.8, 94.0), specificity 90.6% (95% CI: 87.2, 93.3), positive predictive value 84.7% (95% CI: 79.4, 89.0), negative predictive value 94.3% (95% CI: 91.4, 96.4) and accuracy of 90.5% (95% CI: 88.0, 92.8) at 0.403 cutoff point. The calibration test had a *P*-value of 0.165, indicating no significant difference was observed between the observed probability of mortality and the expected probability of mortality (Fig. 5).The brier score of the nomogram was 0.07.

**Fig. 6:**
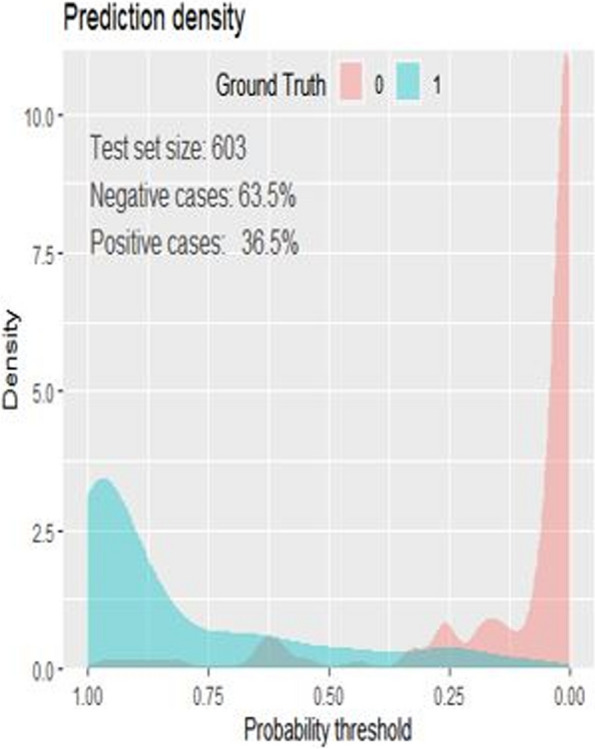
Prediction density plot for mortality prediction nomogram in preterm neonates with sepsis

The performance of the nomogram was also evaluated using prediction density plot. The model hadn’t absolute (100%) discrimination power between positive and negative cases. When the probability threshold approaches to 1, the nomogram can identify the positive cases (those who died) and when the probability approaches to 0 it can identify negative cases (Fig. 6).

#### Mortality risk classification using nomogram

Risk classification was made using Youden’s index” of (max J = 0.81), the corresponding probability cutoff point with this index was 0.403. We dichotomized to low risk (< 0.403) and high risk (≥ 0.403) groups. Out of the total patients 232 (38.5%) were categorized as high risk groups. The incidence of mortality was 4.9% and 87.1% in low risk and high risk groups respectively (Table 7).

**Table 7:** Risk classification of neonatal mortality in preterm neonates with sepsis using nomogram (*n* = 603)

Risk category(a)	Nomogram	
	**Number of patients**	**Incidence of mortality**
Low (< 0.403)	371 (61.5%)	18 (4.9%)
High (≥ 0.403)	232(38.5%)	202 (87.1%)
Total	603 (100%)	220 (36.5%)

^a^Probability of mortality cutoff point identified by Youden’s index method

#### Internal validation

The developed nomogram was internally validated by bootstrapping using 1000 bootstrap samples to determine the degree of overfitting (i.e. models performing better in the development sample than in new sample after bootstrapping). The performance measures of internally validated model were consistent with the nomogram before validation. The AUC of internally validated model was 96.7% (95% CI: 95.6, 97.9) and its corresponding calibration curve *P*-value was 0.165 which indicated the presence of agreement between the observed and predicted probability of mortality across all probability thresholds (bisector).

#### Decision curve analysis

**Fig. 7:**
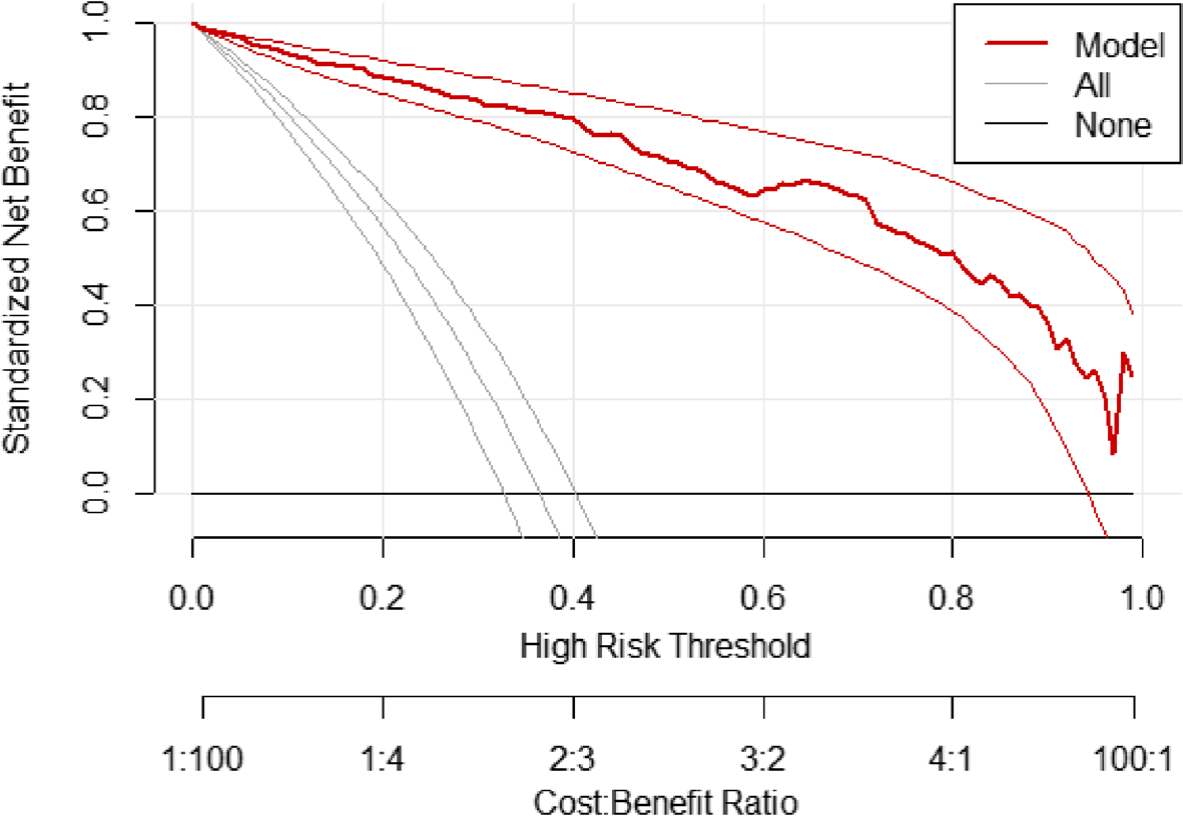
A decision curve plot showing standardized net benefit (y-axis) against threshold probability with corresponding cost–benefit ratios (x-axis). The thick red line represents standardized net benefits of the nomogram across probability thresholds and the accompanying two thin red lines represent the 95% confidence interval; the grey line with its 95% confidence interval represents standardized net benefit of treating all preterm septic neonates in the same way regardless of their mortality risk; the black line represents standardized net benefit of treating none of the preterm septic neonates which is zero

The standardized net benefit of the model was assessed using decision curve analysis. The nomogram had highest net benefit across the entire range of threshold probabilities, which clearly indicate model’s clinical and public health value. Hence, treatment decisions made using the nomogram has a higher net benefit than not treating at all or treating all regardless of their risk threshold (Fig. 7).

## Discussion

This study was aimed to develop a prediction tool (nomogram) that helps to predict neonatal mortality during admission. Prediction models can help health professionals to make clinical decisions through patient risk stratification with the hope of improving patient outcomes and quality of care [33].

The incidence of death in preterm neonates admitted with sepsis was 36.5%. Another study conducted at the same setting showed the incidence of death among preterm neonates was 28.8% [50]. This difference might be due to the included population mortality risk difference. The later study includes preterm neonates regardless of their sepsis status but in our study more risky population (preterm neonates with sepsis) were included. This study finding is also lower than the finding of a study conducted in Felege Hiwot specialized hospital 44.7% [36], Mizan Tepi hospital 65.8% [31] and a study conducted in Ayder and Aksum comprehensive specialized hospitals 45.8% [23]. It is also found higher than a study conducted among neonates referred to comprehensive and specialized hospitals in Amhara regional state 30.6% [6] and Tikur Anbesa Specialized Hospital 29.8% [27]. This higher and lower incidence of neonatal mortality in different settings might be due to geographic variation across the country which resulted from socioeconomic inequalities as established in previous studies [50, 51]. In addition, the variation in quality of care across hospitals in Ethiopia might result this difference [52, 53].

The optimal combination of variables to predict neonatal mortality using nomogram are GA, maternal history of preterm PROM, presence of hypoglycemia, RDS, PNA and NEC comorbidities, total bilirubin, platelet count and KMC.

In this study GA was a significant predictor of mortality. This finding is consistent with other studies [13, 19]. Low gestational age is associated with low birth weight which makes neonates to have higher risk of death because of prematurity related complications [26].

In different studies PNA [24–26, 54, 55] was a significant determinant of mortality. PNA leads to hypoxemia and hypercapnia resulting in central nervous system and end organ damage. The presence of neonatal encephalopathy is considered as an essential causal link between neonatal mortality and PNA [56].

RDS was a significant determinant of mortality which is supported by other studies [23, 27–31, 55]. Insufficient surfactants are usually related with high risk of mortality from hypoxemia and lung injury [26, 54, 57]. NEC increases the risk of mortality in this study which is supported by previous studies [32, 58]. NEC is a multifactorial disease with a significant role for the immunological and gastrointestinal immaturity [59]. Hypoglycemia increased the risk of mortality which is supported by another study [6]. Lack of adipose fat tissue which serve as a source of glucose to adapt the extrauterine life until they maintain through feeding might be a possible explanation for this finding [54].

Neonates who didn’t receive KMC were at higher risk of mortality. This evidence is supported by other findings [22, 23, 60]. KMC prevents hypothermia by reducing body surface area to the external environment and helps easily accessing breastfeed on demand [54].

Decreased platelet count was found associated with increased risk of mortality [61]. The endothelial damage in sepsis activates reticuloendothelial removal of platelets and also with declined level of platelet production.

Level of serum bilirubin is also an established predictor of mortality. During sepsis excessive serum bilirubin concentrations increase risk of acute bilirubin encephalopathy and kernicterus (chronic bilirubin encephalopathy) if not appropriately monitored and treated which increases the risk of mortality from brain toxicity [62, 63].

Preterm PROM increases the risk of neonatal mortality [13]. The role of PROM for preterm birth, sepsis and other intrauterine complications might increase the risk of death [64].

In our study the combination of neonatal birth characteristics, maternal obstetric history, easily obtainable laboratory profiles of the neonate and comorbid medical conditions resulted a prediction nomogram with AUC of 96.7%, which is an excellent model performance according to prediction accuracy classification [65].This nomogram performance was found consistent with a study conducted in Germany which developed artificial neural network for preterm mortality prediction that had AUC of 95.4% [66]. The predictors included were Apgar score, pH from capillary blood gas analysis, gestational age, birth weight, admission condition, congenital malformation and emergency delivery. It is difficult to assess pH from capillary blood gas analysis in our clinical setup. Predictors used for model development should be applicable in low resource clinical settings.

The performance of our nomogram was shown to be better than a preterm infant survival assessment (PISA) prediction model developed in Italy (AUC = 91.4%) [67], a study that develop simplified score to predict mortality in neonates weighing below 2000 g in United Kingdom and Gambia (AUC = 89% and 80% in the United Kingdom and Gambian data respectively) [68]. It was also found better than a study done to derive and validate a risk score for mortality prediction in early neonates in Felege Hiwot Specialized Hospital (AUC = 90.7%) [34] and a study conducted in Debre Tabor referral hospital to predict early neonatal mortality (AUC = 88.7%) [35]. This difference in model performance might be due to the difference in predictors used for model development and the study population. In our study the lethal medical comorbidities like NEC, PNA, hypoglycemia, RDS and low platelet count involved in the model together that might increase the model performance [55, 58]. The other possible explanation might be the population included in this study were more vulnerable groups of neonates for mortality than the study population of the previous studies discussed above.

In our prediction nomogram, based on Youden’s index method using 0.403 as a cutoff probability value has better sensitivity, specificity, positive and negative predictive values as compared to other cutoff probability points. Using this probability cutoff point for mortality prediction improves the model performance and would have good benefit in case of risk stratified treatment.

The benefit of nomogram was also presented in the form of a decision curve. This analysis of examining the real benefit of the model over the treat-all or treat-none conditions, would answer what the discrimination and calibration couldn’t answer. The net benefit of the nomogram was indicated better across threshold probabilities.

The strengths of this study are; firstly we used an adequate number of events per parameter, which helps us to construct the model with enough numbers of predictor variables. The small optimism coefficient identified in the internal validation process indicates the less likely overfitting of the model, and hence it can predict the outcome when applied to an independent set of samples with excellent performance. However it would have been better if it had been done with a prospective data and externally validated. Retrospective studies had limitations like missing important predictors and establishment of temporal relationship. Still retrospective studies are important for prognostic studies in resource limited areas. External validation can increase generalizability beyond the study population. But due to resource, we didn’t externally validate the nomogram. Single centered studies provide less valid findings for external generalizability. Categorizing continuous variables for ease of clinical application might affect the model predictive power and accuracy. We categorize continuous predictors based on their clinical relevance, this would be easy in clinical practice and more applicable than being continuous. Major causes of death during sepsis like intraventricular hemorrhage, disseminated intravascular coagulopathy and multiple organ dysfunction syndrome were not included as predictors because of ascertainment difficulties.

Our study have a public health and clinical implication. Neonatal mortality is a major public health problem and worldwide development agenda. Clinical prediction models that are applied at individual level plays a role in the reduction of overall mortality through giving high emphasis for those at high risk which in turn helps for efficient use of resources since resources are limited mainly in developing countries. Clinicians can use the nomogram to estimate the probability of mortality which directs their decision making process for the patients treatment. Regarding this our study have a great public health importance in the reduction of newborn mortality. Using easily ascertainable characteristics of the neonate, clinicians can predict the probability of mortality which helps them in making treatment decision involving the parents of the neonate. The nomogram risk stratification have excellent specificity and sensitivity, which helps to classify patients as high and low risk of mortality with minimum misclassification risk. The developed model also have higher standardized net benefit across all probability thresholds which clearly indicate its clinical importance. Therefore, the model will be a useful clinical tool for clinicians to apply in their decision-making process. Furthermore, this study would help SDG and ENAP goal achievements. The main strategy for the implementation of the prediction model is ensuring that the use of the prediction model by the targeted healthcare professionals and patients will have a positive effect on both clinical decision making and prognostic outcomes [69]. The possible challenge might be incorporating the model in the clinical care practice which should pass many steps including external validation, impact assessment and inclusion of the model in the national policy.

## Conclusion

The developed model, which have an excellent level of accuracy and good calibration can be applied to predict neonatal mortality in preterm neonates admitted with sepsis. The model was found beneficial in clinical practice as it was assured by net benefit analysis.

## Data Availability

The data used for this study is available from the corresponding author on reasonable request.

## Abbreviations

AGA: Appropriate for Gestational Age
ANC: Antenatal Care
APH: Antepartum Hemorrhage
AUC: Area Under the Curve
BW: Birth Weight
CI: Confidence Interval
ENAP: Every Newborn Action Plan
GA: Gestational Age
HDP: Hypertensive Disorders of Pregnancy
KMC: Kangaroo Mother Care
LASSO: Least Absolute Shrinkage And Selection Operator
NEC: Necrotizing Enterocolitis
PNA: Perinatal Asphyxia
PROM: Premature Rupture of Membrane
RDS: Respiratory Distress Syndrome
ROC: Receiver Operating Characteristics
SDG: Sustainable Development Goal
SGA: Small For Gestational Age
SSA: Sub-Saharan Africa
